# Human Leukocyte Antigens in Pregnancy and Preeclampsia

**DOI:** 10.3389/fgene.2022.884275

**Published:** 2022-04-27

**Authors:** Omonigho Aisagbonhi, Gerald P. Morris

**Affiliations:** Department of Pathology, University of California San Diego, San Diego, CA, United States

**Keywords:** pregnancy, preeclampsia, HLA, HLA-G, HLA-C

## Abstract

Preeclampsia is a pregnancy-induced hypertensive disorder, the pathophysiology of which includes underlying maternal cardiovascular disease, deficient spiral artery remodeling during placenta development, and inflammatory immune responses at the maternal-fetal interface. Human leukocyte antigens (HLA) are major histocompatibility complex molecules essential for the recognition of foreign antigens that is central to immune defense against pathogens and critical determinants for the immune system discriminating between self and non-self tissues, such as in transplantation. Pregnancy represents a naturally existing “transplantation”, where the maternal immune system must be immunologically tolerant to the developing fetus which is 50% allogeneic. It is then unsurprising that HLA also influence normal pregnancy and pregnancy complications including preeclampsia. Here we review the role of classical and non-classical HLA molecules in influencing normal physiologic function during pregnancy and describe the association of HLA with pathophysiology in preeclampsia.

## Introduction

**FIGURE 1:**
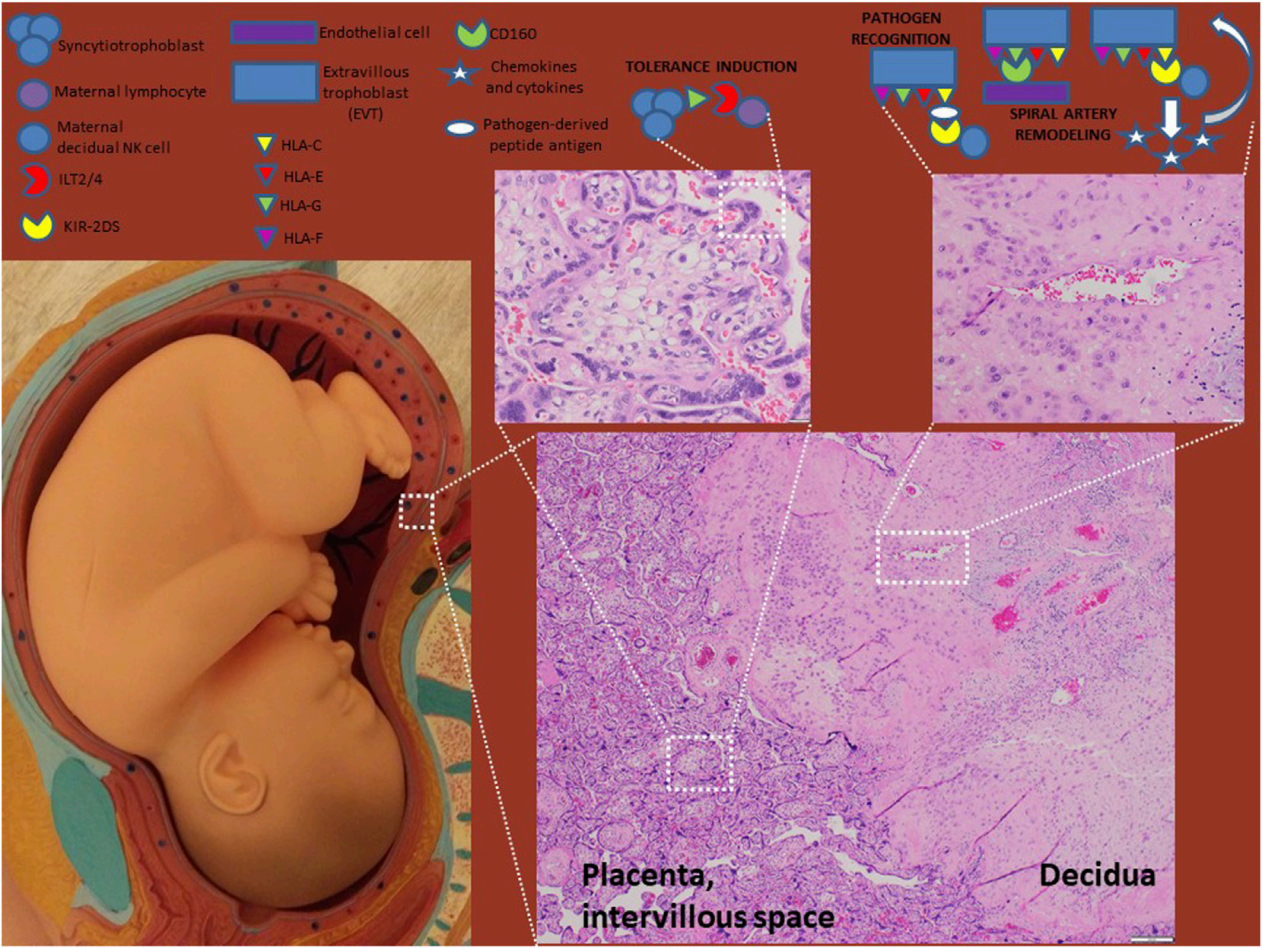
HLA molecules in normal pregnancy. HLA-G-expressing syncytiotrophoblasts interact with the maternal systemic immune system at the intervillous space to induce tolerance. EVTs express HLA-C, -E, -F and–G and interact with maternal decidual immune cells and endothelial cells to induce tolerance and remodel spiral arteries. Optimal placentation occurs when HLA-C engages activating KIR receptors (e.g. KIR2DS1/5) resulting in release of chemokines and cytokines (e.g. GM-CSF and IL-8) that enhance EVT invasiveness. HLA-C also plays a role in pathogen recognition and antigen presentation.

Pregnancy can be viewed as a naturally-occurring allograft, with a semi-allogeneic fetus requiring access to maternal circulation for provision of nutrients and oxygenation during development. In contrast to the robust immune responses typically elicited by allogeneic stimulation, the maternal immune system does not mount an inflammatory response to the fetus under normal physiology. This immunologic tolerance is facilitated by the development and function of the placenta. The placenta is a fetal organ that develops to become the interface between which maternal and fetal cells interact (Figure 1). Placental trophoblast cells are the primary fetal cells directly exposed to the maternal immune system (Moffett and Loke, 2006). Villous trophoblasts, comprised of an inner layer of cytotrophoblasts and an outer layer of syncytiotrophoblasts, are directly in contact with maternal blood and maternal systemic immune system at the intervillous space. Extravillous trophoblasts (EVTs) migrate from the cytotrophoblast shell through decidual stroma and remodel maternal decidual spiral arteries into low resistance, high conductance dilated vessels without smooth muscle, which enables optimal flow of blood for oxygen exchange to support placental and fetal growth (Burton, et al., 2009). Interaction between fetal trophoblast cells and maternal immune cells at the placental interface are essential for regulating these remodeling processes and maintaining maternal immunologic tolerance to the fetus.

## Preeclampsia

Preeclampsia is a pregnancy-induced hypertensive disorder defined as new-onset hypertension and proteinuria or new-onset hypertension and end-organ (heart, lung, brain, kidney, liver) dysfunction, occurring between 20 weeks of gestation and 6 weeks post-partum (Bouter and Duvekot, 2020). Preeclampsia is relatively common, affecting as many as 4–10% of all pregnancies worldwide. In the most severe cases, preeclampsia can be fatal, with maternal end-organ failure. Despite the significant risks of morbidity and mortality from preeclampsia, no singularly effective cure exists, with treatment relying on early detection and administration of antihypertensive medications to bridge the mother and fetus to delivery. The efficacy of this approach is limited, with preeclampsia persisting as one of the five leading causes of maternal mortality worldwide (Say, et al., 2014). The incidence and clinical outcomes in preeclampsia vary by geographic region and maternal ethnicity, suggesting both environmental and genetic components contribute to the pathophysiology.

Emerging evidence suggests that rather than having a single cause, preeclampsia is a multifactorial syndrome that can result from multiple distinct pathophysiologic processes. Using unsupervised clustering of aggregate microarray datasets (first in 173 patient samples, including 77 with preeclampsia and later in a larger dataset of 330 samples), 3 molecular clusters/subclasses of preeclampsia have been proposed (Table 1) (Leavey, et al., 2015; Leavey, et al., 2016). Preeclampsia samples in cluster 1 showed normal placental histopathology and the birthweights of delivered infants were appropriate for gestational age. At the molecular level, there was no significant difference in gene expression by cells in the placenta between preeclampsia cases from cluster 1 and controls without preeclampsia. Thus, it was reasoned that underlying maternal factors, unrelated to the placenta, are the likely etiology of preeclampsia in cluster 1 patients. Clusters 2 and 3 had more clinically significant presentations, with higher maternal blood pressures, higher proteinuria and uric acid levels. Placentas in cluster 2 demonstrated classic preeclampsia-associated findings (small for gestational age, distal villous hypoplasia, with syncytial knots and infarcts) and were significantly more likely to be associated with infants that were small for gestational age (SGA) and/or had a clinical diagnosis of intrauterine growth restriction. Genes involved with hormone secretion, response to nutrient, redox activity and response to hypoxia/angiogenesis were over-represented in cluster 2. Preeclampsia placentas in cluster 3 showed increased rates of massive perivillous fibrin deposition, a placental lesion associated with maternal anti-fetal rejection (Romero, et al., 2013; Gundogan, et al., 2010), and increased expression of genes associated with allograft rejection, immune and inflammatory responses.

**TABLE 1:** Summary of clinical, pathologic and molecular findings in the 3 proposed etiologic clusters of PE–maternal, canonical and immunologic (Leavey, et al., 2016).

Findings	Maternal Preeclampsia (Cluster 1)	Canonical Preeclampsia (Cluster 2)	Immunologic Preclampsia (Cluster 3)
**Clinical**	Infant birthweight appropriate for gestational age	Infant birthweight small for gestational age	Infant birthweight small for gestational age
**Gross and histopathologic**	Placenta weight appropriate for gestational age	Placenta weight small for gestational age	Placenta weight small for gestational age
	Normal histology	Maternal vascular malperfusion (distal villous hypoplasia, syncytial knots, infarcts)	Massive perivillous fibrin deposition
**Molecular**	No significant difference from normotensive controls	Over-representation of genes associated with hypoxia/angiogenesis, hormone secretion, nutrient and redox activity	Increased expression of genes associated with allograft rejection, immune and inflammatory responses
**Proposed etiology**	Maternal cardiovascular disease (unrelated to placenta)	Deficient spiral artery remodeling during placenta development	Maternal-fetal incompatibility manifested in the placenta

Based on these findings, three etiologic categories for PE have been proposed—1) maternal preeclampsia caused by maternal factors unrelated to the placenta; 2) canonical preeclampsia caused by deficient spiral artery remodeling and 3) immunologic preeclampsia caused by maternal-fetal incompatibility. Considering preclampsia as a syndrome with at least 3 different etiologic categories begins to explain why the disease has different clinical (mild vs severe; early vs late-onset) and pathologic manifestations (small placenta with evidence of maternal vascular malperfusion vs increased perivillous fibrin deposition with or without an associated chronic villitis, vs normal sized placenta without significant pathology) (Roberts, et al., 2021). Within this context of multi-modal pathophysiology it becomes possible to consider the influence of genetics on preeclampsia.

## Human Leukocyte Antigen

The human leukocyte antigen (HLA) system is a cluster of >100 genes regulating immune function located on the short arm of chromosome 6p21.3 (Shiina, et al., 2009). Most notably, the HLA locus contains genes encoding the *HLA* genes, human orthologs of Major Histocompatibility Complex (MHC) genes. HLA/MHC proteins’ primary function is to present antigens to T lymphocytes, enabling recognition of foreign antigens by the cellular arm of the adaptive immune system (Neefjes, et al., 2011). The defining genes of the HLA locus include the classical HLA class Ia genes *HLA-A*, *HLA-B*, and *HLA-C*, encoding the HLA-A, HLA-B, and HLA-C proteins which present peptide antigens to CD8^+^ cytotoxic T cells, and the HLA class II genes *HLA-DRA1*, *HLA-DRB1*, *HLA-DRB3/4/5*, *HLA-DQA1*, *HLA-DQB1*, *HLA-DPA1*, and *HLA-DPB1* which encode the proteins forming the HLA-DR, HLA-DQ, and HLA-DP molecules that present peptide antigens to CD4^+^ helper T cells, including immunosuppressive regulatory T cells (Tregs). The genes encoding the classical HLA molecules are the most genetically diverse genes in the human genome, with over 25,000 defined allelic variants (Robinson, et al., 2020), with even more alleles being identified regularly. The extensive nucleotide sequence variability translates to protein polymorphisms that influence the ability of HLA molecules to bind and present antigens as well as interact with leukocyte antigen receptors. This diversity underpins the robust immune responses of T lymphocytes to allogeneic tissues, with HLA protein sequence diversity both directly affecting the interaction of T cell antigen receptors with the HLA molecule and indirectly stimulating T cell responses via differential presentation of endogenous tissue antigens (Felix and Allen, 2007).

In addition to presentation of peptide antigens to T lymphocytes, HLA-C (and some HLA-A, and HLA-B molecules, though these are not expressed by EVTs) is involved in the regulation of NK cell activity. Class I HLA molecules interact with NK cells in a (mostly) peptide-independent manner through a combination of activating and inhibitory signals initiated by the binding of Killer Immunoglobulin Receptor (KIR) molecules (Parham, 2005). These interactions involve multiple different KIR molecules, each with differential ability to interact with HLA-C and other ligands, and each containing different intracellular signaling motifs. There are 17 identified KIR genes, each of which have 30–230 known genetic allotypes, located in the Leukocyte Receptor Complex (LRC) region on chromosome 19q13.4 (Barrow and Trowsdale, 2008; Robinson, et al., 2013). KIR genes can be broadly divided into activating “DS” receptors, containing short intracellular domains with activating signaling motifs, and inhibitory “DL” receptors, containing longer intracellular domains with inhibitory signaling motifs. An individual’s KIR gene repertoire varies both by allelic variation as well as gene content, with the presence or absence of specific KIR genes being the primary determinant of the ability of NK cells to interact with HLA-C. NK cell recognition of HLA-C is determined by which amino acid is expressed at position 80 of the HLA-C molecule. In terms of KIR interaction, HLA-C molecules are divided into 2 groups, with the HLA-C1 group having asparagine (Asn) at position 80 while the HLA-C2 group contains lysine (Lys) at position 80. HLA-C1 group molecules interact with activating KIR2DS2 and inhibitory KIR2DL2 and KIR2DL3 receptors while HLA-C2 group molecules interact with activating KIR2DS1 and inhibitory KIR2DL1 receptors (Parham, 2005).

Beyond the classical HLA molecules, the *HLA-E*, *HLA-F*, and *HLA-G* genes (HLA class Ib genes) encode non-classical HLA class I proteins HLA-E, HLA-F and HLA-G which can present a relatively limited set of peptide and non-protein antigens to leukocytes, including T lymphocytes, non-classical T cells, and Natural Killer (NK) cells (Carosella, et al., 2015; Grant, et al., 2020; Persson, et al., 2020). Additionally, some interactions between non-classical HLA molecules and leukocyte receptors are antigen-independent, with recognition of the HLA molecule by receptors being deterministic for inducing immune cell response. The interactions between non-classical HLA molecules and their associated receptors can provide either stimulatory or inhibitory signals to immune cells, depending on the specific HLA-receptor combination. In contrast to classical HLA genes, the genes encoding non-classical HLA molecules have limited genetic diversity (Robinson, et al., 2020). The effects of these limited polymorphisms on interaction with leukocyte receptors is incompletely defined, though there is evidence that this genetic variation, particularly when located in untranslated regulatory regions, can result in differences in non-classical HLA expression (Gobin and van den Elsen, 2000; Strong, et al., 2003; Hviid, et al., 2003). This genetic diversity can result in selective activation of immune cells in response to allogeneic cells, either by interaction with allogeneic HLA molecules that provide a stimulatory signal or via the absence of interactions required to provide inhibitory signals.

## Human Leukocyte Antigen in Normal Pregnancy

Interactions of HLA molecules with T cells and NK cells are essential determinants of immune function and discrimination between self- and non-self. As such, HLA molecules have a critical role in the interactions between fetal trophoblasts and maternal immune cells. The maternal immune system has to maintain a balance between immunologic tolerance for the semi-allogeneic fetus and the ability to respond to pathogens to prevent infection. HLA-C and the non-classical HLA class Ib molecules are important for normal pregnancy, where they play roles in modulating maternal immune responses to the semi-allogeneic fetus, spiral artery remodeling and pathogen recognition. HLA expression differs between villous trophoblasts and EVTs (Figure 1). While neither villous trophoblasts nor EVTs normally express HLA-A, HLA-B, or HLA class II molecules, EVTs express HLA-C, HLA-E, HLA-F and HLA-G while villous trophoblasts only express soluble forms of HLA-G (Ishitani, et al., 2003; Apps, et al., 2009). It is unclear whether trophoblast expression of HLA molecules changes during gestation. Increased expression of HLA-E and HLA-F by EVTs from the first to second to third trimester has been observed (Shobu, et al., 2006); however, a contrasting study found HLA-E expression limited to the first trimester and expression of HLA-C, HLA-F and HLA-G decreased as gestation progressed (Hackmon, et al., 2017). This restricted and potentially dynamic expression of HLA molecules influences their ability to regulate immune cell function at the maternal-fetal interface.

### Placental Human Leukocyte Antigen and Protective Immune Responses

The most obvious physiologic function of HLA molecules at the maternal-fetal interface is to present antigens from infectious pathogens, enabling the maternal immune system to mediate protective cellular immune responses. Fetal EVTs, like all other mature cells, co-dominantly express both chromosomal copies of *HLA* genes. Thus, the semi-allogeneic fetal EVTs express maternally-derived HLA-C, enabling self-MHC-restricted maternal T cells to respond to presented foreign antigens. HLA-C expressed by fetal EVT are also able to present pathogen-derived peptide antigens to maternal NK cells via interactions with KIR2DS1, KIR2DS2, and KIR2DS4 activating receptors, stimulating NK cell-mediated responses to infection (Crespo, et al., 2016; Naiyer, et al., 2017; Sim, et al., 2019). HLA-E is also capable of presenting a limited set of pathogen-derived antigens, including peptides derived from cytomegalovirus (CMV) and Human immunodeficiency virus (HIV), which can cause significant morbidity and mortality in neonates (Grant, et al., 2020). HLA-E is capable of stimulating NK cells via the activating KIR2DS1 and NKG2C receptors (Braud, et al., 1998; Lee, et al., 1998). HLA-F is similarly capable of stimulating NK cells via KIR3DS1 (Garcia-Beltran, et al., 2016; Burian, et al., 2016). Thus, despite the restricted set of HLA molecules expressed by trophoblasts, the maternal immune system is capable of detecting infection and mediating innate and adaptive cellular immune responses. Co-dominant expression of HLA-C alleles also results in expression of likely genetically dissimilar paternal HLA-C by EVTs, which presents the challenge of potential alloreactive responses by maternal immune system.

### Placental Human Leukocyte Antigen and Inhibition of Inflammatory Immune Responses

The absence of immunologic rejection of the semi-allogeneic fetus is not due to a lack of ability of the maternal immune system to recognize the foreignness of fetus. Indeed, multiple studies have demonstrated the existence of fetal antigen-reactive T cells, both at the placental interface and in circulation (Tilburgs, et al., 2010; Lissauer, et al., 2012; van der Zwan, et al., 2018). Inhibition of inflammatory immune responses and promotion of immunologic tolerance at the maternal-fetal interface instead relies upon multiple physiologic mechanisms. Expression of the inhibitory co-stimulatory PD-L1 (Nagamatsu, et al., 2009) by EVTs, production of the immunosuppressive metabolite indoleamine-2-3-dioxygenase (IDO) (Munn, et al., 1998), and expression of immunosuppressive cytokines such as IL-10 and TFG-β (Guzeloglu-Kayisli, et al., 2009) all act to directly inhibit the activity of inflammatory and cytotoxic lymphocytes in the placental microenvironment. The non-classical HLA class Ib molecules HLA-G, and HLA-E have also been reported to directly inhibit cytotoxic and pro-inflammatory T cells and NK cells at the placenta. HLA-G interacts with decidual NK cells, T cells, B cells, and myeloid cells via KIR2DL4 receptor on NK cells, ILT2 receptor on T cells, B cells, and some NK cells, and ILT4 receptor on myeloid cells (Colonna, et al., 1998; Rajagopalan and Long, 1999; Shiroishi, et al., 2006). HLA-G is capable of inhibiting antigen-specific and alloreactive cytotoxic T cell responses *in vitro* (Le Gal, et al., 1999; Kapasi, et al., 2000). Expression of HLA-G is also sufficient to prevent NK cell-mediated cytolysis (Chumbley, et al., 1994; Pazmany, et al., 1996). HLA-G can also mediate immunosuppressive function in the absence of direct cell-cell contact. HLA-G is unique among HLA molecules, with the existence of alternatively spliced mRNA forms which produce seven different HLA-G protein isoforms (Ishitani and Geraghty, 1992; Fujii, et al., 1994). The isoforms include conventional membrane-bound G1-G4 isoforms and G5-G7 secreted soluble (sHLA-G) proteins. sHLA-G molecules have inhibitory effects on T cells and NK cells similar to those of membrane-bound HLA-G. sHLA-G is produced by trophoblasts (Solier, et al., 2002), suggesting a role for HLA-G in regulating maternal immune responses. Indeed, both membrane-bound or soluble HLA-G inhibits CD4^+^ and CD8^+^ T cell responses and NK cell-mediated cytolysis of allogeneic cytotrophoblasts *in vitro*, indicating the functional relevance of this inhibition at the maternal/fetal interface (Rouas-Freiss, et al., 1997; Kostlin, et al., 2017). HLA-E has been shown to be similarly protective against NK cell-mediated lysis via interactions with activating NKG2C receptor on NK cells, inhibitory CD94/NKG2A receptors on NK cells, and CD8 on T cells (Braud, et al., 1998; Lee, et al., 1998).

Trophoblast-expressed HLA-G also influences the generation and persistence of CD4^+^FoxP3^+^ Tregs. Tregs are important mediators of immunologic tolerance, capable of regulating the responses of immune cells and preventing pathologic inflammatory responses (Sakaguchi, et al., 2020). The regulatory function of maternal Tregs is essential at the maternal/fetal interface to prevent maternal alloimmune response and immunologic rejection of the fetus (Tsuda, et al., 2021). Maternal Tregs are specific for fetal antigens and activated via their antigen receptor by peptide antigens presented by HLA class II molecules. While trophoblasts do not express HLA class II molecules, maternal Tregs are exposed to fetal antigens presented by HLA II molecules on antigen-presenting cells such as dendritic cells and macrophages in the placental microenvironment. The relative importance of Tregs in suppressing maternal alloreactive immune responses at the maternal-fetal interface can be demonstrated by the observation that Tregs are increased in pregnancies where maternal and fetal *HLA-C* genotypes are mismatched (Tilburgs, et al., 2009). This suggests that the Treg response must be increased as a necessary response to increased allogeneic stimulation by the fetus. Several studies have indicated that placental tissue, specifically EVTs expressing HLA-G, promote differentiation of CD4^+^ T cells into Tregs (LeMaoult, et al., 2004; Tilburgs, et al., 2015; Svensson-Arvelund, et al., 2015). This Treg induction is associated with inhibition of alloreactive T cell responses, demonstrating the potential mechanism by which fetal HLA-G contributes to immunologic tolerance via Treg induction.

### Human Leukocyte Antigen and Placental Development

A more unique role for HLA molecules in pregnancy occurs during the process of physiologic conversion, whereby maternal spiral arteries are remodeled into low-resistance, high-conductance vessels, which is necessary for the growth and function of the placenta. During this process, EVTs interact with local maternal decidual NK cells, and stimulate them to aid in tissue remodeling during fetal development. NK cell activity is critical during physiologic conversion, and is regulated by interaction with HLA-C, HLA-G, and HLA-E. Interactions between HLA-C and activating KIRs stimulate NK cells to secrete chemokines and cytokines such as GM-CSF, IL-8, and interferon-inducible protein 10, that promote trophoblast migration and ultimately contribute to spiral artery remodeling during normal placentation (Hanna, et al., 2006; Xiong, et al., 2013). HLA-G also promotes NK cell activity contributing to trophoblast invasion and spiral artery remodeling. sHLA-G5 stimulates trophoblast invasion *in vitro* by binding KIR2DL4 and LILRB1 (a.k.a. ILT2), activating the ERK signaling pathway and increasing production of tissue remodeling-associated proteases uPA and MMPs as well as proangiogenic cytokines and chemokines (Rajagopalan, et al., 2006; Guo, et al., 2013; Fu, et al., 2017; Gamliel, et al., 2018). During spiral artery remodeling, HLA-G may also interact with endothelial cells directly, as HLA-G1 induced endothelial cell apoptosis via binding endothelial CD160 receptor (Fons, et al., 2006). Roles for HLA-E and HLA-F in regulation of NK cell activity during physiologic conversion have not been demonstrated, though both HLA-E and HLA-F are expressed by invading EVTs and interact with receptors on NK cells.

## Human Leukocyte Antigen in Placental Pathology

### Human Leukocyte Antigen in Maternal Preeclampsia

The 3 pathophysiologic clusters of preeclampsia proposed based on placental gene expression include preeclampsia resulting from maternal factors, deficient placental development, and immunologic incompatibility (Leavey, et al., 2015; Leavey, et al., 2016). Of these categories, maternal preeclampsia, considered as a consequence of underlying maternal factors such as cardiovascular disease and obesity, is the least likely to be directly influenced by placental HLA genetics. However, it should be noted that some underlying maternal autoimmune diseases that increase risk for preeclampsia have susceptibilities that have been linked to HLA. An example is systemic lupus erythematosus (SLE), which has been associated with a 1.91 relative risk for preeclampsia in a meta-analysis (Bundhun et al., 2017). Increased SLE susceptibility has been linked to HLA-DRB1*15 alleles across multiple ethnic populations (Selvaraja et al., 2022). Ankylosing spondylitis, a disease whose susceptibility is strongly linked to HLA-B*27 (Ebrahimiadib et al., 2021) has similarly been associated with poor pregnancy outcomes (Jakobsson et al., 2016). Other autoimmune diseases such as type 1 diabetes mellitus and rheumatoid arthritis also have increased risks conferred by HLA class II haplotypes (Ebrahimiadib et al., 2021; Enczmann et al., 2021). Nevertheless, as HLA-B and class II HLA molecules are not normally expressed by placental trophoblasts, the mechanism by which maternal autoimmune diseases lead to poor pregnancy outcomes like preeclampsia and preterm birth needs to be further elucidated.

### Human Leukocyte Antigen in Immunologic Preeclampsia/Maternal-Fetal Incompatibility or Tolerance Failure

The concept of the fetus as a semi-allogeneic graft is based upon the fact that the fetus is 50% of maternal genetic origin and 50% paternal genetic origin. HLA genes are typically inherited *en bloc* as a unit containing all of the genes from a single chromosome 6p21 region, inheriting 1 maternal set or haplotype and 1 paternal haplotype. Given the extensive genetic diversity of *HLA* genes, a fetus is expected to be genetically dissimilar from the mother for HLA genes, most commonly haploidentical or mismatched for 1 allele of each of the classical HLA genes. The influence of maternal-fetal HLA mismatches in immunologic preeclampsia is unclear. Retrospective analysis of oocyte donation pregnancies found pregnancy losses and preeclampsia were associated with increased numbers of HLA gene mismatches between maternal-fetal pairs (Lashley, et al., 2015; van Bentem, et al., 2020). However, studies on HLA mismatches and preeclampsia in spontaneously conceived pregnancies have yielded varied and sometimes contradictory findings with no clear evidence for an association between HLA dissimilarity between the mother and fetus and the incidence of preeclampsia (Hoff, et al., 1992; Biggar, et al., 2010; Triche, et al., 2014; Van’t Hof et al., 2021).

A lack of clear association between HLA mismatches and preeclampsia may reflect limitations in the analyses performed. Separation of the effects of individual HLA genes on preeclampsia has similarly mixed results, with demonstration of selective influence by either HLA class I (Lashley, et al., 2015; van’t Hoff, et al., 2021) or HLA class II (Triche, et al., 2014; van Bentem, et al., 2020; van’t Hoff, et al., 2021) genes. Of the class I genes, only *HLA-C* has been demonstrated to have statistically confident influence independent from other HLA mismatches (van’t Hoff, et al., 2021). This likely reflects the fact that fetal trophoblast cells do not express *HLA-A* or *HLA-B*, but do express *HLA-C*, making it the only classical HLA class I molecule available to interact with maternal immune cells. Dissection of the effect of *HLA-C* mismatches indicates that fetal expression of paternal alleles in the C2 group are associated with increased risk for preeclampsia and placental immune lesions (Hiby, et al., 2004; Hiby, et al., 2010; Schonkeren, et al., 2012; Larsen, et al., 2019; van’t Hoff, et al., 2021). Presumably, this association is driven by differential interaction of C2 group molecules with either activating or inhibitory KIR molecules. However, this association is complicated by population-specific variability in maternal KIR genetics, which determine the repertoire of NK cell receptors available to interact with HLA-C molecules. Maternal inhibitory KIR AA genotype has been shown to be associated with increased risk of preeclampsia in Europeans from the United Kingdom, East-Africans and Han Chinese and maternal activating KIR B genotypes (KIR2DS1 in Europeans and Ethiopians, KIR2DS5 in East Africans and KIR2DS2/3/5 in Han Chinese) have been shown to be protective against preeclampsia (Hiby et al., 2004; Hiby et al., 2010; Long et al., 2015; Nakimuli et al., 2015; Kelemu et al., 2020). However, a Danish study of fetal HLA-C and maternal KIR genotypes in severe preeclampsia found no significant association between maternal KIR AA and fetal HLA-C2 alleles and severe preeclampsia risk (Larsen et al., 2019). Studies evaluating the expression patterns of HLA-C and activating versus inhibitory KIRs in the placentas of patients with mild vs severe preeclampsia in contrast to those without preeclampsia across different ethnic groups may be informative.

Potential effects for maternal/fetal HLA class II mismatches, specifically *HLA-DRB1* and *HLA-DQB1*, with preeclampsia are less straightforward, as neither villous nor extravillous trophoblasts normally express HLA class II molecules. However, aberrant expression of HLA-DR by syncytiotrophoblasts has been observed in placentas of women with preeclampsia (Small, et al., 2017; Tersigni, et al., 2018). This likely reflects an expected physiologic response to placental inflammation, as MHC class II gene expression is regulated by the Class II Transcription Activator (CIITA) transcription factor, which is upregulated by the inflammatory cytokine interferon γ (Steimle, et al., 1994). The pathophysiologic consequences of this dysregulation are undefined, though it may represent a mechanism for aberrant stimulation of CD4^+^ helper T cells or dysregulation of Treg function.

The non-classical HLA class I molecules HLA-F and HLA-G have also been identified as potentially dysregulated in preeclampsia. While both *HLA-F* and *HLA-G* have relatively limited genetic diversity compared to classical HLA genes, both have identified expression variants, either via prematurely terminating null variants or regulatory elements in non-coding regions (Robinson, et al., 2013)**.** While reduced expression variants of HLA-F have not been associated with preeclampsia, the rs1362126, rs2523405, and rs2523393 variants have been associated with reduced fertility (Langkilde, et al., 2020). The importance of HLA-F in inhibiting NK cell-mediated cytotoxic responses was postulated as a potential mechanism for this association. The influence of HLA-G on preeclampsia is better described. Multiple studies have observed decreased HLA-G expression, both by fetal cells in the placenta as well as soluble forms in circulation, in patients with preeclampsia is associated with impaired maternal-fetal immune tolerance (Yie, et al., 2004; Steinborn, et al., 2007; Vianna, et al., 2016). Decreased HLA-G expression in preeclampsia has been associated with reduced frequencies of regulatory T cells and increased expression of pro-inflammatory cytokines (Vianna, et al., 2016; Wedenoja, et al., 2020). The mechanism by which HLA-G is decreased in preeclampsia is undefined. A number of studies have suggested that a fetal 14bp insertion/deletion polymorphism may account for decreased HLA-G expression and increased preeclampsia risk (O’Brien, et al., 2001; Hylenius et al., 2004; Hviid, et al., 2004; Rokhafrooz, et al., 2018), though other studies have not found the same association (Pabalan, et al., 2015; Nilsson, et al., 2016). Down-regulation of *HLA-G* expression via hypermethylation of the promoter has been proposed as an alternative mechanism for downregulation of HLA-G in preeclampsia (Tang, et al., 2015). Epigenetic dysregulation of HLA-G is of significant interest as it represents a mutable and potentially actionable target for intervention.

### Human Leukocyte Antigen in Deficient Spiral Artery Remodeling

Defective spiral artery remodeling during physiologic conversion is well-recognized as an important etiology of preeclampsia (Brosens, et al., 1972; Lim, et al., 1997; Fisher, 2015). As described above, spiral artery remodeling is dependent on interactions between HLA-C, HLA-G, and HLA-E molecules on EVTs and receptors on decidual NK cells and vascular endothelial cells. The association of fetal HLA-C2 genotypes with preeclampsia has been proposed to result from a lack of appropriate NK cell activation during spiral artery remodeling (Hiby, et al., 2004; Hiby, et al., 2010; Nakimuli, et al., 2015; Long, et al., 2015). Reduced expression of HLA-G by EVTs has been similarly associated with preeclampsia (Colbern, et al., 1994; Hara, et al., 1996; Goldman-Wohl, et al., 2000). However, it is difficult to disentangle the observed effects of HLA-C and HLA-G in spiral artery remodeling from their roles in inhibiting inappropriate inflammatory responses in the majority of studies to date. An interesting possibility is that the differential roles (anti-inflammatory versus spiral artery remodeling) may be related differential interactions with maternal immune, stromal and endothelial cells and their various receptors.

### Anti-Human Leukocyte Antigen Antibodies and Preeclampsia

Circulating antibodies directed against paternal-derived HLA antigens can be detected in normal pregnancies (first described by Van Rood et al., 1958 and Payne and Rolfs, 1958). However, whether these antibodies contribute to pregnancy complications like preeclampsia is controversial. A meta-analysis found no consistent effect of anti-HLA class I or class II antibodies on pregnancy outcome (Lashley et al., 2013).

## Summary

**FIGURE 2:**
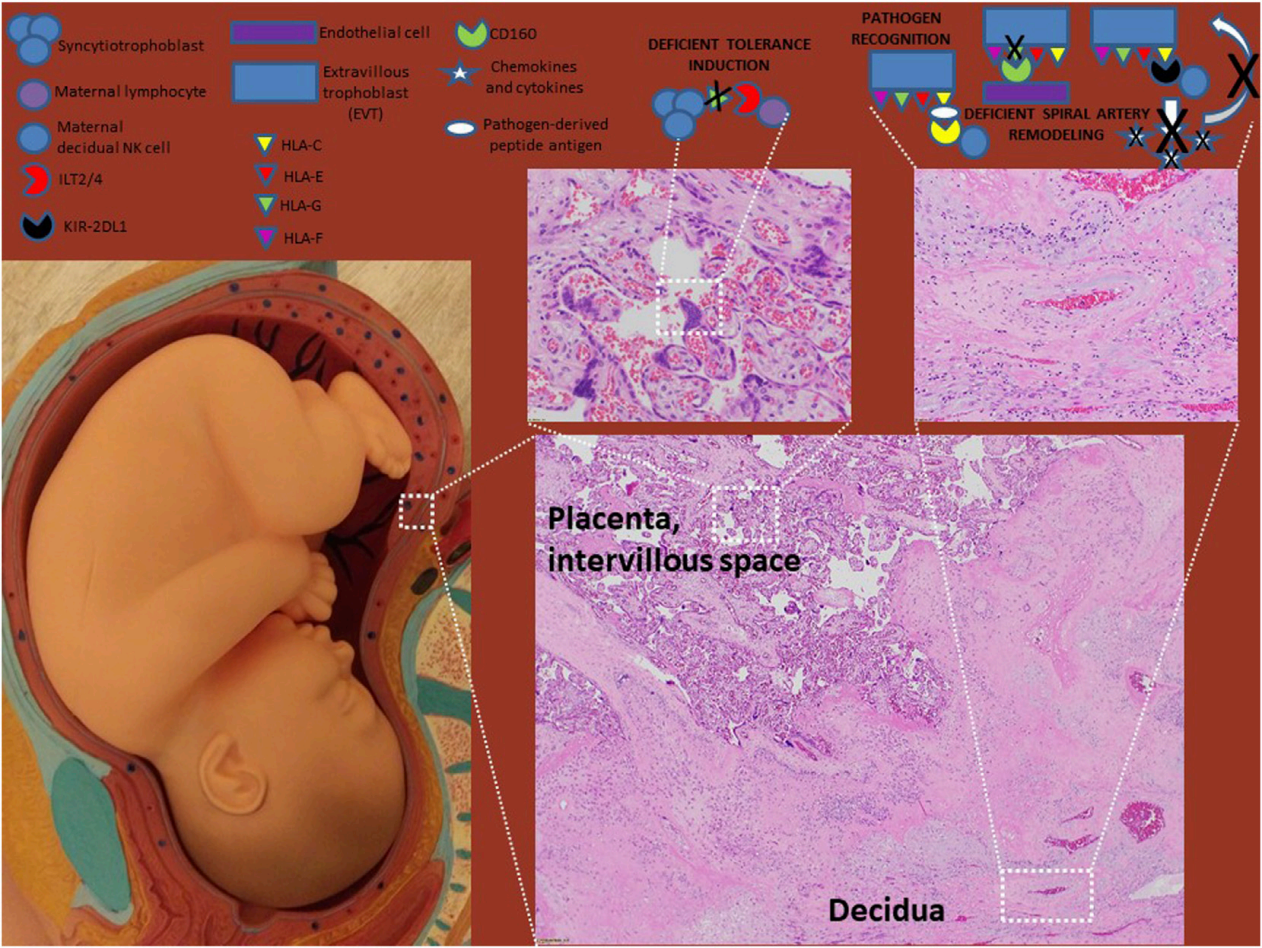
HLA molecules in preeclampsia. HLA-G levels are decreased in preeclampsia which evidence suggest result in defects in tolerance induction and spiral artery remodeling. Though disputed by some studies, most studies suggest that HLA-C engaging inhibitory KIR receptors on NK cells (such as KIR2DL1) also leads to defects in spiral artery remodeling as failed NK cell release of pro-migratory chemokines and cytokines limits EVT invasion. The roles of HLA-E and HLA-F in preeclampsia need to be investigated further.

This review has summarized current understanding of the roles HLA molecules play in pregnancy and preeclampsia (Figures 1, 2; Table 2). Given the central role of HLA molecules in immune function, their near-ubiquitous expression, and the considerable amount of genetic diversity in *HLA* genes directly affecting HLA function, it is not surprising that HLA is commonly identified as associated with diseases having known genetic factors. Of the HLA molecules (HLA-C, HLA-E, HLA-F, and HLA-G) expressed by placental trophoblasts, HLA-C and HLA-G are the most studied, with well-defined roles both in ensuring maternal tolerance of the semiallogeneic fetus and in tissue remodeling required for spiral artery development for adequate perfusion of the developing placenta. The functions of HLA-E and HLA-F in pregnancy and preeclampsia are less well-described and warrant further investigation.

**TABLE 2:** Summary of the roles HLA-C, E, F and G play in normal pregnancy and how their dysregulation contributes to preeclampsia.

HLA Molecule	Role in Normal Pregnancy	Dysregulation in Preeclampsia
HLA-C	Expressed by EVTs	Some studies suggest that women with preeclampsia are more likely to have inhibitory KIR genotypes whereby interactions between HLA-C and KIR on NK cells fails to stimulate release of pro-migratory chemokines/cytokines and thus result in defective spiral artery remodeling
	Presents pathogen-derived peptides to maternal T and NK cells and activates their cytotoxic responses for infection control	
	Interaction between HLA-C and KIRs stimulates NK cells to release chemokines and cytokines promoting trophoblast migration and spiral artery remodeling/physiologic conversion	
HLA-E	Expressed by EVTs	Dysregulation/dysfunction in preeclampsia needs to be further investigated
	Presents a limited set of viral pathogen-derived peptides to maternal T and NK cells and activates their cytotoxic responses for infection control	
	Protects trophoblasts from NK cell-mediated lysis via interactions with CD94/NKG2A receptors	
HLA-F	Expressed by EVTs	Dysregulation/dysfunction in preeclampsia needs to be further investigated
	Genetic variants causing reduced expression associated with decreased fertility, supporting as-yet undefined role in regulating NK cell activity at maternal-fetal interface	
HLA-G	Expressed by both villous trophoblasts and EVTs	Decreased placental and circulating HLA-G levels are observed in preeclampsia and are associated with deficient spiral artery remodeling, decreased numbers of Tregs and increased expression of pro-inflammatory cytokines
	Promotes maternal tolerance of the semi-allogenic fetus by inhibiting cytotoxic activity of maternal T and NK cells, and promoting the generation and persistence of Tregs	
	Promotes physiologic conversion/spiral artery remodeling by stimulating NK cells to release pro-migratory and proangiogenic chemokines and cytokines. Also interacts with endothelial cells via CD160	

A significant limitation of the studies on maternal/fetal HLA mismatches to date is the treatment of all dissimilarity at the genetic level as equivalent. However, there may be distinctions among these mismatches, with certain differences having differential functional impact. The classification of *HLA-C* mismatches into C1 and C2 groups is an example of functional classification of mismatches. However, HLA-C1/C2 classification only relates to regulation of NK cells and does not address the canonical role of classical HLA molecules in the presentation of antigens to T cells. We propose that it may be informative for future analyses to examine HLA differences in this function, using measures of structural dissimilarity and antigen binding (Sidney, et al., 2008; Chowell, et al., 2019). Similarly, there may be maternal *HLA* genetic factors (such as HLA class II genotypes) influencing the maternal immune response that have yet to be appreciated.

The recent proposal classifying preeclampsia as resulting from 3 distinct pathophysiologic pathways (Leavey, et al., 2015; Leavey, et al., 2016) also raises the possibility that any influence of *HLA* genotypes on preeclampsia may be obfuscated by not having separated analyses into more well-defined categories. Indeed, an absence of an effect of genes regulating immune function such as *HLA* would not be surprising in non-immune mediated pathology, and thus may be underestimated by not distinguishing immune-mediated and non-immune-mediated mechanisms for preeclampsia. We propose that this distinction in future studies may more clearly reveal genetic associations and functional roles for the *HLA* genes and help define genetic risk factors in preeclampsia.
